# Comparing pH differential and methanol‐based methods for anthocyanin assessments of strawberries

**DOI:** 10.1002/fsn3.2065

**Published:** 2021-11-05

**Authors:** Toktam Taghavi, Hiral Patel, Reza Rafie

**Affiliations:** ^1^ Agricultural Research Station Virginia State University Petersburg VA USA; ^2^ Cooperative Extension Virginia State University Petersburg VA USA

**Keywords:** chloroform fraction, flavonoids, methanol extract, method development, organic solvent, pigments

## Abstract

Anthocyanins are a group of water‐soluble polyphenolic pigments found primarily in flowers, vegetables, and fruits. These pigments play critical roles in plant and human health. Spectrophotometric methods are a simple and inexpensive way to quantify anthocyanins in plant tissues. Two main spectrophotometric methods have been developed, organic solvent‐based, and pH differential methods. Both of these methods are subject to interference from light‐absorbing impurities and need to be optimized for different matrixes of different plant materials. Eight methods have been tested in this experiment to quantify anthocyanins in strawberry fruits. Six organic solvent‐based methods tested methanol, chloroform‐methanol, and MgO in different ratios. The other two methods were pH differential method and a combination of organic solvent‐based and pH differential method. Two methods used organic solvents (methanol and chloroform‐methanol) were the best in extracting anthocyanin from strawberry fruits. Adding MgO increased the pH of the extract and was less efficient in anthocyanin extraction. All other methods had lower anthocyanin yield compared with methanol and chloroform‐methanol methods and are not recommended for strawberry fruit anthocyanin extraction.

## Graphical abstract

Two organic solvents, methanol and chloroform‐methanol, were the best in extracting anthocyanin from strawberry fruits. MgO decreased anthocyanin yield. All other methods had lower anthocyanin yield compared with methanol and chloroform‐methanol methods.

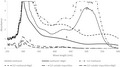

## 1 INTRODUCTION

Anthocyanins are water‐soluble polyphenolic pigments found primarily in fruits, vegetables, and flowers. Anthocyanins belong to a class of organic molecules called flavonoids, a group of polyphenol compounds. Flavonoids originate from phenylalanine, the product of the shikimate pathway (Broeckling et al., 2012) and malonyl‐CoA the product of the acetic acid pathway. The combination between both pathways leads to the formation of flavonoids, the most abundant group of phenolic compounds in nature. The basic chemical structure of the flavonoids is based on two six‐carbon rings linked by a three‐carbon unit (C6–C3–C6), also known as the chalcone structure. Ring B and the 3‐carbon unit are derived from the shikimic acid pathway, and the carbons in ring A are derived from malonic acid acetyl‐coenzyme A (acetyl‐CoA) and three molecules of malonyl‐CoA (Zuiter, 2014). The first step is catalyzed by chalcone synthase (CHS). This enzyme catalyzes the stepwise condensation of 4‐coumaroyl‐CoA (a by‐product of phenylalanine) and three molecules of malonyl‐CoA to yield anthocyanidins and anthocyanins in a multi‐step process (Desjardins, 2008).

Anthocyanins play critical roles in the survival and physiology of plants. Anthocyanins have a photo‐protective role and are used to attract pollinators for seed dispersal (Gallik, 2012). Anthocyanins are present in the cytosol and/or stored in the vacuole of the plant cells and trap excessive solar radiation (Steele et al., 2009) by absorbing UV before it reaches UV‐sensitive targets such as chloroplasts and other organelles (Alexieva et al., 2001). Anthocyanins also act as a plant stress indicator because their biosynthesis is initiated due to stress factors such as pests, drought, nutrient deficiency, and extreme temperature (Steele et al., 2009).

Anthocyanins have high antioxidant features and help the human body in disease prevention and the healing process. They have been linked to reduced coronary heart disease risks (Martín et al., 2017), increased visual acuity, and anticancer properties (Pavlidou et al., 2018).

Due to the various protective roles anthocyanins play in plants and human health, research into the cellular effects of anthocyanins has intensified. Therefore, accurate quantification of anthocyanin content is increasingly crucial, and many research groups are working to determine the concentration of anthocyanins in fruits, vegetables, and their by‐products (Gallik, 2012).

Several methods for anthocyanin quantification have been developed. While nondestructive methods such as spectral reflectance are still under development (Steele et al., 2009), traditional analytical techniques are the main methods to measure the concentration of anthocyanins. In the past, chromatographic and capillary electrophoresis methods such as paper and thin‐layer chromatography were used (Trikas et al., 2016). More commonly today, anthocyanins are generally quantified by spectrophotometric methods in plant tissues (Solovchenko et al., 2001).

Two main methods have been developed to assess anthocyanins. The first method uses organic solvents (methanol, ethanol, or acetone) to extract anthocyanins in an acidic environment (Lindoo & Caldwell, 1978). The second method uses structural changes in anthocyanins in two different pH (1 and 4.5) to assess anthocyanin (pH differential method; Lee et al., 2005). These methods are subject to interference from light‐absorbing impurities present in the extracts (Solovchenko et al., 2001).

Of the common organic solvents, methanol performs better than ethanol as a solvent for the extraction of anthocyanins (Trikas et al., 2016). It is the most widely used extraction solvent when acidified with HCl (Silva et al., 2017). Solovchenko et al. (2001) claimed that chlorophylls and carotenoids interfere during light absorption and should be removed for anthocyanin assessment. They concluded the application of chloroform would eliminate the content of light‐absorbing impurities that interfere during light absorption and should be removed for anthocyanin assessment in apple peel samples. They used Folch et al. (1957) method to separate lipid‐ and water‐soluble components of extracts between the chloroform and water‐methanol fractions (Solovchenko et al., 2001). They also claimed that the addition of MgO during extraction will prevent pigment degradation in apple peels and will give a higher absorbance. Neff and Chory (1998) also used chloroform: methanol in a different ratio during anthocyanin extraction from Arabidopsis leaf samples. Neff and Chory (1998) also suggested chloroform and methanol for the extraction of anthocyanins. While Solovchenko et al. (2001) did not use water, Neff and Chory (1998) added water to the extraction buffer.

The second main method is the pH differential method. This method is based on the reversible structural changes in anthocyanins due to manipulating the samples’ pH (Mazza et al., 2004). Anthocyanin color is dependent on the pH‐dependent positive charge on the C ring of the molecule. The C ring carries a positive charge, and the molecule is pigmented at a pH of 1.0 and neutralized and colorless at a pH of 4.5 and higher (Gallik, 2012). Therefore, at a pH of 1.0, the molecule strongly absorbs light between 460 and 550 nm; however, it is colorless at a pH of 4.5. Thus, the difference in absorbance at 520 nm of the pigment is proportional to the concentration of pigment (Lee et al., 2005) and permits accurate and rapid measurement of the total anthocyanins, even in the presence of polymerized degraded pigments and other interfering compounds (Giusti & Wrolstad, 2001). Gauche et al. (2010) suggested combining two methods. They extracted anthocyanin of grape skin with acidified methanol and then measured it by the pH differential method.

Due to the pigment's complex chemistry, the extraction method needs to be optimized for the different matrixes (Silva et al., 2017). Researchers have used different methods for different fruit crops with unique matrixes.

Canuto et al. (2016) developed a liquid chromatography method for the assessment of anthocyanin in strawberries. Liu et al. (2008) compared HPLC and spectrophotometric methods for the analysis of anthocyanins in strawberries and blueberries. Gauch et al. (2010) used a combination of the organic solvent and pH differential methods to assess the anthocyanins in grapes. However, there is no comparison between the three main methods (organic solvent, pH differential, and their combination) and little or no justification as if any of these three methods works better for a specific type of fruit.

Strawberries are a rich source of nutrients and anthocyanin for the human diet. Several methods have been used to extract and assess anthocyanins of strawberries, such as liquid chromatography (RPLC‐DAD, Canuto et al., 2016), HPLC (Liu et al., 2008), and high‐performance liquid chromatography‐electrospray ionization‐mass spectrometry (Karaaslan & Yaman, 2017).

Spectrophotometric methods such as pH differential (Benchikh et al., 2020; Tonutare et al., 2014) and organic solvents were also used to assess anthocyanins in strawberries (Liu et al., 2008). The two main methods reported for strawberries are acidified methanol (Garcia‐Viguera et al., 1998; Liu et al., 2008) and pH differential method (Lee et al., 2005; Tonutare et al., 2014). Unlike liquid chromatography, spectrophotometric methods do not require expensive equipment or highly technical skills. However, it is not clear what method of spectrophotometric anthocyanin assessment is best suited for strawberries.

We compared the organic solvents, pH differential, and their combination to extract and assess anthocyanins from strawberry fruits. The objective was to identify a spectrophotometric method with a higher extraction yield of anthocyanins and ease of use. Methanol‐based methods and the pH differential method were used to assess the anthocyanins of a bulk strawberry fruit sample. The uniform bulk strawberry sample eliminated the sample differences and ensured variations in yield reflect the efficacy of the extraction method. The methanol‐based methods and the pH differential method were chosen because they are both accurate, simple, and rapid methods of measuring monomeric anthocyanin content. To increase the anthocyanin yield (reduce impurities and pigment degradation), the effect of chloroform and MgO (Solovchenko et al., 2001) was also tested.

## 2 MATERIALS AND METHODS

### 2.1 Chemicals

Cyanidin‐3‐glucoside (C3G), sodium acetate, potassium chloride, magnesium oxide (MgO), Hydrochloric acid, Methanol, and chloroform were purchased from Sigma. Mega pure water was obtained from Thermo Scientific Barnstead Smart2Pure 3 LPH UV/UF system.

### 2.2 Instrumentation

Equipment used in this experiment were UV/Visible spectrophotometer, Genesys 150 UV‐Vis spectrophotometer connected to Visionlite 5 software, shaker, incubator (precision refrigerated incubator, all from Thermo Fisher Scientific), and blender (Magic Bullet 600‐Watt).

### 2.3 Standard and blank preparation

Stock standard solution of Cyanidin‐3‐Glucoside (C3G) was made by dissolving 4 mg/L in Methanol: HCl (0.1%) and stored at −25°C until further use. C3G was used as an internal standard, and 1 ml was used instead of a sample in all methods (Canuto et al., 2016). Internal standards were treated in the same manner and at the same time as samples. Distilled water was used as a blank in the spectrophotometer for all methods because, according to Giusti and Wrolstad (2001), the buffer's absorbance is nil at the measured wavelengths.

### 2.4 Sample preparation

Five hundred grams of strawberry fruits were ground using the blender to form a fine puree and create a more representative sample. Five grams of the strawberry puree were weighed and stored in small containers with lids and frozen quickly (−30°C) to reduce oxidative modifications until further use.

### 2.5 Anthocyanin assessment methods

For the assessment of anthocyanins in strawberries, total of eight methods were tested. Six methods are based on organic solvent extraction; one was the pH differential method (Lee et al., 2005), and the other was a combination of both (Gauche et al., 2010).

The first method was the methanol method suggested by Lindoo and Caldwell (1978). Fifteen millilitre of methanol:water:concentrated HCl (80:20:1) was added to 5 g of frozen strawberry samples and incubated at 4°C in the dark on the shaker. Two incubation periods of 24 hr and 48 hr were tested. At the end of the incubation period, the homogenates were centrifuged at 4°C, 8,382 *g* for 15 min. The supernatant was then removed and stored in 15 ml tubes at −25°C. The absorbance of anthocyanin was measured by the spectrophotometer at 530 and 657 nm (Alexieva et al., 2001; Lindoo & Caldwell, 1978). The anthocyanin concentrations of the two incubation periods were analyzed using SAS software (SAS 2015, version 9.4) in a completely randomized design.

In the second method (Solovchenko et al., 2001), anthocyanins were extracted by adding 10 ml of methanol: concentrated HCl (0.1%) to the 5 g strawberry samples. The homogenates were incubated on the shaker in the dark at 4°C for 24 hr and centrifuged at 4°C, 8,382 *g* for 15 min. The supernatant was removed and stored in 15 ml tubes at −25°C for further study.

The third method followed the Solovchenko et al. (2001) and Folch et al. (1957) method and was very similar to the second method, except that chloroform was added to remove impurities. Ten millilitre of chloroform‐methanol (2:1 v/v, acidified with 0.1% HCl) mixture was added to the strawberry samples. The homogenates were incubated on the shaker in the dark at 4°C for 24 hr. The homogenate was centrifuged at 4°C, 8,382 *g* for 15 min to separate the chloroform and methanol‐water fractions. Both fractions were removed and stored in 15 ml tubes at −25°C for further study.

For the fourth method, 0.1 g of MgO was mixed with the 5 g frozen strawberry samples, and the samples were treated the same as the second method (Solovchenko et al., 2001). The homogenates were incubated on the shaker in the dark at 4°C for 24 hr. The extract was then centrifuged at 4°C, 8,382 *g* for 15 min, and the supernatant was removed and stored in a 15 ml tube at −25°C.

In the fifth method, 0.1 g of MgO was mixed with the 5 g frozen strawberry samples, and the samples were treated the same as the third method (Solovchenko et al., 2001). The homogenates were incubated on the shaker in the dark at 4°C for 24 hr. The extract was then centrifuged at 4°C, 8,382 *g* for 15 min, and the supernatant was removed and stored in a 15 ml tube at −25°C. The fourth and fifth methods investigated the addition of MgO to prevent pigment degradation.

For all the methods mentioned above (methods 2–5), the absorbance of the supernatant (A) was read at 530 and 657 nm before and after storing at −25°C for one week. Only the readings before storage are presented here. The pH of the extracts from methods 2–5 (methanol and chloroform‐methanol with and without MgO) was also measured.

For the sixth method (Neff and Chory (1998), 15 ml methanol, 10 ml water, 0.15 ml HCL, 25 ml chloroform were added to the 5 g frozen samples, incubated at 4°C for 24 hr in the dark on the shaker and centrifuged at 4°C, 8,382 *g* for 15 min. The supernatant was then removed and stored in a 15 ml tube at −25°C. The absorbance (A) was read at 530 and 657 nm. Anthocyanin concentration in methods mentioned above (method 1–6) was determined by the following formula (Jayakumar et al., 1999) and was given as A/g fresh fruit tissue, where TA = total anthocyanin, A = absorbance at 530 and 657 nm, V = volume of extract (ml), and *M* = fresh mass of the sample (g).

$$\text{TA}=\frac{A530‐0.3A657*V}{M}$$

Strawberry fruit anthocyanin concentration was also measured by the pH differential method (seventh method; Lee et al., 2005). The frozen strawberry samples (5 g) were mixed thoroughly with 20 ml buffer pH 1.0 (0.025 M potassium chloride) and then incubated for 20 min at room temperature and centrifuged at 4°C, 8,382 *g* for 15 min. The supernatant was then removed, and the absorbance was read at 520 and 700 nm. The 5 g frozen strawberry samples were then combined with 20 ml buffer pH 4.5 (0.4 M sodium acetate buffer) and incubated and centrifuged similarly (Gu et al., 2018). The absorbance of the supernatant was measured at the same wavelengths. A modified formula was used to compare the pigment concentration of different methods:

$$\text{TA}=\frac{A*V}{M}$$

where *A* = (A520 nm − A700 nm) pH 1.0 − (A520 nm − A700 nm) pH 4.5; *V* = volume of extract (ml) and *M* = fresh mass of the sample (g).

In the Gauche et al. (2010) method, 5 g of strawberry samples was extracted in darkness for 24 hr, with 20 ml of 0.1% HCl in methanol at 4°C. The crude extract obtained was centrifuged at 4°C, 8,382 *g* for 15 min. The supernatant was removed, and half of the original volume (12.5 ml) was concentrated under vacuum at 35°C until reaching about 50% of the methanol volume. The quantification of total anthocyanin concentration in the concentrated extract was measured according to the pH differential method (Lindoo & Caldwell, 1978).

The eight methods had different solvent/mass ratios (V/M). The ratio was integrated into the formulas to eliminate the differences in the ratios and make the methods comparable. All the experiments had four replicates, and the experiment was repeated twice. The average data were used to calculate anthocyanin concentration. Wavelength scanning of the first replicates was used to create the absorbance spectrum (absorption between 250 and 600 nm for every 2 nm) for different wavelengths of the extracted anthocyanins.

## 3 RESULTS

There were eight methods tested using frozen strawberry fruits. All methods will be compared with the first method (methanol method) to facilitate data comparison. In all methods, 5 g of strawberry samples from a uniform puree was frozen and used. Therefore, differences in absorbance and anthocyanin concentration are not related to differences in the strawberry samples; rather, the ability of the specific method to extract anthocyanin, prevent pigment degradation, and remove light‐absorbing impurities.

**Figure 1:**
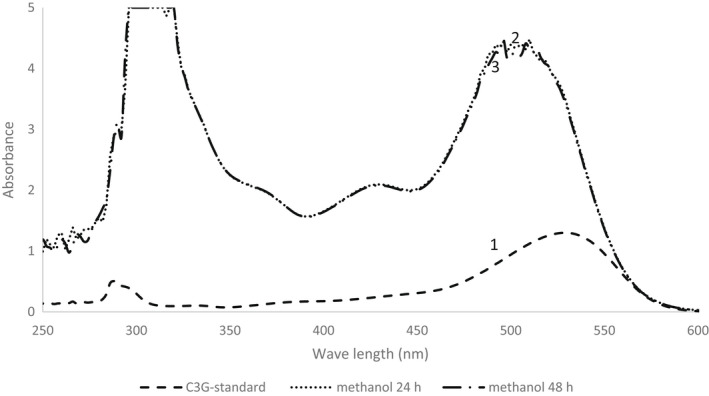
The absorption spectrum of (1) Cyanidin‐3‐Glucoside standard solution used as internal control, strawberry anthocyanin extracted by methanol method (2) after 24 hr incubation, and (3) after 48 hr incubation

Cyaniding‐3‐glucoside (C3G) was used as the internal standard, as it is the most common form of anthocyanin in nature. The standard anthocyanin, C3G, had the maximum absorbance at 530 nm (Figure 1:1), confirming the maximum absorbance of 530 nm for anthocyanins in assessed samples. There was also a peak between 300 and 320 nm in all extracts, which did not interfere with anthocyanins, but might be the phenols or flavor compounds.

In the first method (methanol method), two incubation periods (24 and 48 hr) were tested. Almost the same amount of anthocyanin was extracted from both incubation periods (11.6 & 11.8 A/gFW, respectively, Table 1), and there were no significant differences between them. Also, their absorption spectra were very similar in all wavelengths, as well as the anthocyanin absorption area of 530 nm (Figure 1:2,3). Therefore, for the rest of the experiments, 24 hr of incubation time was used.

**Table 1:** Anthocyanin concentration of frozen strawberries measured by organic solvent and pH differential methods

Method tested	Anthocyanin concentration A/gFW	*SD*	C3G Conc.	Formula
Methanol 24 hr (1)^a^	10.0	0.4	1.3	(A_530_−0.3A_657_ × 15)/5
Methanol 48 hr (1)	10.2	0.3	‐b	(A_530_−0.3A_657_ × 15)/5
Chf‐methanol without MgO (3)	10.1	0.03	0.9	(A_530_−0.3A_657_ × 10.15)/5
Chf‐methanol‐Neff (6)	9.0	0.2	0.8	(A_530_−0.3A_657_ × 25)/5
methanol without MgO (2)	8.5	0.1	1.3	(A_530_−0.3A_657_ × 15)/5
pH differential (7)	5.9	8.2	0.9	(A_520_−A_700_)pH1‐(A_520_‐A_700_)pH4.5 × 25/5
Combined‐Gauche (8)	5.4	0.4	0.9	(A_520_−A_700_)pH1‐(A_520_‐A_700_)pH4.5 × 25/5
Chf‐methanol with MgO (5)	2.5	0.6	0.2	(A_530_−0.3A_657_ × 10.15)/5
methanol with MgO (4)	1.8	0.4	0.2	(A_530_−0.3A_657_ × 15)/5

Abbreviations: *SD*, standard deviation; A, absorbance, C3G, Cyaniding‐3‐glucoside used as the internal standard.

^a^ (1) numbers in the brackets refer to the method number; Eight methods were tested: (1) methanol method (Lindoo & Caldwell, 1978) incubated for 24 and 48 hr, (2) methanol method (Solovchenko et al., 2001), (3) chloroform‐methanol method (Solovchenko et al., 2001), (4) method 2 with MgO, (5) method 3 with MgO, (6) chloroform‐methanol (Neff & Chory, 1998), (7) pH differential method (Lee et al., 2005), and (8) combination of methanol and pH differential methods (Gauche et al., 2010).

^b^ C3G was not incubated for 48 hr.

**Figure 2:**
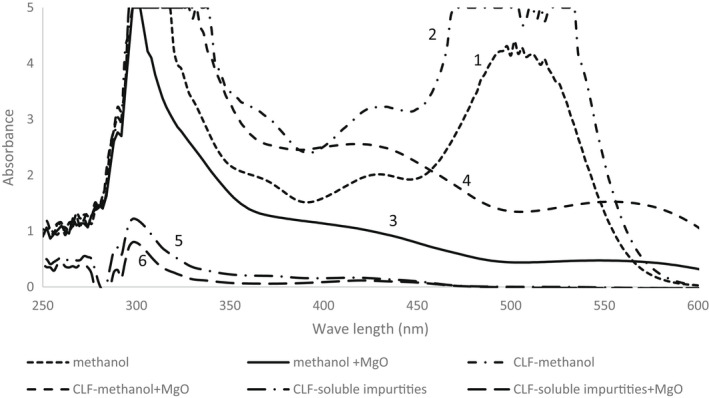
The absorption spectrum of strawberry anthocyanin extracted by (1) methanol extract after 24 hr, (2) chloroform‐methanol extract, (3) methanol extract in samples with MgO, (4) chloroform‐methanol extract in samples with MgO, (5) chloroform fraction (CLF) showing chloroform‐soluble impurities, and (6) chloroform fraction showing chloroform‐soluble impurities in samples with MgO

In the second and third methods (adapted from Solovchenko et al., 2001), the addition of chloroform was compared with methanol in removing light‐absorbing impurities (i.e., chlorophylls, carotenoids, and lipids). The absorption spectrum of the chloroform‐methanol method was higher than the methanol method (Figures 2:1,2). However, this could be due to the lower volume of the extraction buffer. Ten millilitre of methanol:water: HCl (80:20:1) was used in the first method and 5.15 ml methanol: HCl (33.3:1) for the second method. The chloroform‐methanol method also had higher anthocyanin concentration compared with the methanol method (Table 1). However, it is not very clear if the higher absorption of the chloroform‐methanol method is due to its lower solvent/mass ratio or other factors (i.e., removing impurities). Therefore, the absorption spectrum of the chloroform fraction was also measured (Figure 2:5). However, chloroform fraction did not carry impurities that absorb light in the range of anthocyanin absorption (about 530 nm), but it removed impurities that absorb light in the range of 250–350 nm. Therefore, higher absorption of the chloroform‐methanol method is not related to the role of chloroform in removing impurities that interfere with anthocyanin absorption and should be related to the solvent/mass ratio or other parameters.

To test the hypothesis that MgO prevents pigment degradation, MgO was added to the strawberry samples in both methods (methanol and chloroform‐methanol methods). The results have shown that the addition of MgO in both methanol and chloroform‐methanol methods decreased the absorbance of the samples significantly at 530 nm (Figure 2:3,5). MgO increased the pH of the extraction buffer considerably and faded the pink color of anthocyanin (Akkarachaneeyakorn & Tinrat, 2015) and converted it to purple/blue quinine color, with low absorption at 530 nm (Favaro et al., 2018). The pH of the samples with MgO was 7.3, and the ones without MgO were 1.5 in both methanol and chloroform‐methanol methods. In the methanol method, the average anthocyanin concentration was 1.8 in samples with MgO compared to 8.5 without MgO, and in the chloroform‐methanol method, the concentrations were 2.5 and 10.1, respectively (Table 1). Adding MgO under‐estimated the amount of anthocyanin. The low absorbance is mostly due to the increased pH of the extracted anthocyanin, which reduces the absorbance at 530 nm. To further study the effect of MgO, the pH of the extracted anthocyanin in the presence of MgO was decreased by adding one drop of concentrated HCl to the extract. The color of the extracted supernatant changed to purple, and the absorbance at 530 nm increased significantly. However, the absorbance was still lower compared to the methods without MgO (data not presented). Again, the chloroform fraction absorbed light in the range of 250–350 nm, and these impurities should not disturb the anthocyanin assessment.

**Figure 3:**
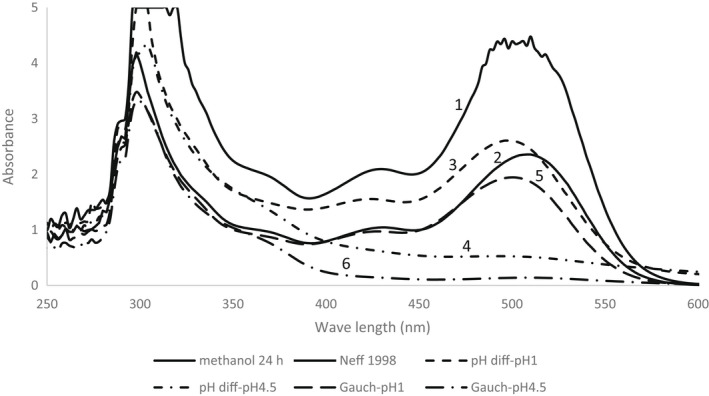
The absorption spectrum of strawberry anthocyanin extracted by (1) methanol extract after 24 hr, (2) chloroform‐methanol extract as in Neff and Chory (1998), (3) pH differential method, pH1, (4) pH differential method, pH4.5, (5) methanol extract followed by pH differential method, pH 1, as in Gauche et al. (2010), and (6) methanol extract followed by pH differential method, pH 4.5

The Neff and Chory (1998) method had a very similar extraction buffer to the chloroform‐methanol method. The difference is that Neff and Chory (1998) added water during extraction, while the chloroform‐methanol method did not. This method had lower anthocyanin absorption than the methanol method (Table 1) and a smaller absorption spectrum (Figure 3:1,2). Therefore, it is not recommended for strawberry anthocyanin assessment.

The pH differential method has two absorption spectrums, one for pH 1.0 and the other 4.5. This method is based on the differences between absorption at 520 nm between the colorless structure of anthocyanins at pH 4.5 and the highest absorption for pigmented structure at pH 1.0. The anthocyanin concentration in the pH differential method (pH 1.0) was lower than the methanol method (Table 1, Figure 3:1,3), meaning less anthocyanin was extracted. However, it is a speedy method with only 20 min incubation time. The extract was a little hazy in the pH differential method, even after it was centrifuged at 8,382 *g* twice. In this method, measuring the absorbance at 700 nm corrected for the haze (Lee et al., 2005). However, we have stored the supernatants of all methods at −25°C to be used in further experiments. After a freeze‐thaw process, deposits formed in the supernatant of the pH differential method, the haze increased, and the anthocyanin absorption decreased (data not shown). In contrast, the anthocyanin extract in all other methods did not change after a thawing process. Therefore, if the storage of the anthocyanin extract is needed, the pH differential method is not a suitable method for the assessment of anthocyanins in strawberries.

In the Gauche et al. (2010) method, the anthocyanin absorption was lower than the methanol method and in the range with pH differential methods (3–5 & 3–6). This method had a lengthy process. First, anthocyanins had to be extracted by the methanol method. After incubation and centrifugation, the supernatant volume should be reduced by vacuum. Then, the anthocyanin concentration to be measured by the pH differential method. Therefore, for these two reasons, longer processing time and lower anthocyanin yield, it is not suitable for anthocyanin assessments in strawberries.

## 4 DISCUSSION

All the tested methods used the same approach to extract anthocyanins from plant tissues, which was to soak and subsequently extract anthocyanins with solvents. Anthocyanins are usually extracted in polar, organic solvents such as water, methanol, ethanol, or acetone at a composition of 70%–80% in water. Of the common solvents, methanol performed better than ethanol for the extraction of anthocyanins in strawberries (Karaaslan & Yaman, 2017) and wine (Trikas et al., 2016); therefore, it was used in this experiment. Acidified solutions are often used because they make it easier for the anthocyanins to pass through the cellular membranes and be obtained by the extraction solution. Low concentrations of strong acids, such as HCl, were the best options (Trikas et al., 2016) and were used in this experiment.

Several parameters affect the anthocyanin yield, such as sample type, incubation time, temperature, solvent type, and solvent/mass ratio. A uniform sample was used for all the tested methods to eliminate the effect of sample type. The eight methods had different solvent/mass ratios (Volume/Mass). The ratio was integrated into the formulas to eliminate the differences. In this experiment, the increased contact time between solvent and tissue from 24 to 48 hr did not increase the yield because the diffusion from solid particle to liquid had reached its equilibrium. The time frame in which equilibria was reached varied according to several other conditions (i.e., temperature and solvent type). The temperature was constant for all the experiments, and 24 hr was chosen for incubation time, except for the pH differential method, where incubation time was 20 min. Therefore, the solvent type was the only factor that contributed to the anthocyanin yield.

Both methanol and chloroform‐methanol extraction methods (methods 1–3) extracted a considerable amount of anthocyanins from frozen strawberry fruits. They gave us clear supernatant with the highest concentrations. Solovchenko et al. (2001) mentioned that methanol extracts were often turbid, and preliminary chloroform extraction of the cuticular lipids of the apple peel disks eliminated this turbidity. However, in strawberry fruit extracts, the methanol extract was clear, and no turbidity was observed. The higher absorbance spectrum in the chloroform‐methanol method could be related to the lower solvent/mass ratio compared with the methanol method. Researchers claimed that pheophytin and other chlorophyll degradation products might significantly contribute to the absorption of extracts at 530 nm, and chloroform will remove them (Solovchenko et al., 2001). However, the absorbance of the chloroform fraction was very low at 530 nm, which is the absorbance wavelength for anthocyanins. In strawberry fruits, correcting for the presence of pheophytin and other products of chlorophyll degradation did not deem necessary, as the high absorbance of the scanning spectrum for chloroform fraction was near the UV region (300–320) and should not interfere with anthocyanin absorbance. This peak could reflect aromatic compounds such as coumarins (Taniguchi & Lindsey, 2018) and quinones (Howard et al., 2014), as strawberry seems to be a rich source of them.

It is suggested that anthocyanins may be stabilized by adding metallic ions (i.e., Mg) to plant tissues by hampering the oxidation of the quinoidal base (Silva et al., 2017). However, it was not proven to be beneficial in extracting anthocyanins from strawberry fruits. Adding MgO to the samples increased the pH considerably and did not increase the absorbance compared to samples without MgO in both methanol and chloroform‐methanol methods. Typically, anthocyanins are more stable under acidic conditions, and pH values above 7 lead to their degradation (Akkarachaneeyakorn & Tinrat, 2015; Silva et al., 2017). A strong acidic environment is needed to change the structure of anthocyanin to develop the color of anthocyanins. Solovchenko et al. (2001) have added acid to the assay mixture of apple peels to the point that changed the pH of the extract to a very acidic environment. However, when we added HCl to the extract, the extracts' absorption was still lower than samples without MgO. Therefore, we concluded that the addition of MgO did not improve the anthocyanin extraction of strawberries and did not affect pigment degradation.

Neff and Chory (1998) method could not improve the anthocyanin yield compared with the methanol method. The extraction buffer uses the same ingredients as the chloroform‐methanol method; however, it has higher chloroform to methanol ratio. Neff and Chory (1998) used it for anthocyanin extraction of *Arabidopsis* leaves where higher chlorophyll content exists. The higher ratio may help remove the chlorophylls of leaves; however, it was not beneficial in strawberry fruit.

The pH differential method has been used extensively by food technologists and horticulturists to assess the quality of fresh and processed fruits and vegetables. The method can be used to determine total monomeric anthocyanin concentration, based on the structural change of the anthocyanin chromophore between pH 1.0 and 4.5. In our experiment, there was a haze that did not completely disappear with increasing the centrifugation time. The haze increased and formed a deposit if the anthocyanin extract was exposed to a freeze‐thaw process. Therefore, this method did not seem reliable for strawberry anthocyanin extraction, especially if the extract needs to be stored and re‐measured. None of the other researchers have mentioned the haze as a persistent issue in strawberry anthocyanin extraction. However, this method was quick, with a short incubation time (20 min). Therefore, if time is a constraint and a large number of samples need to be tested, this method can extract anthocyanin and assess them quickly with reasonable accuracy.

Gauche et al. (2010) method for “Cabernet Sauvignon” grape was a long process and had three steps, extraction of anthocyanin with methanol, reducing the extraction volume by vacuum, and reading the anthocyanin concentration by the pH differential method. There were two drawbacks to this method. First, the process was longer than other methods, which may have increased anthocyanins degradation due to longer processing time and higher temperatures. Second, the anthocyanin yield was lower than the methanol method, therefore, deemed unsuitable for strawberry fruit anthocyanin assessment.

## 5 CONCLUSION

It is well documented that several factors, such as sample type, incubation time, temperature, solvent components, and their ratio, pH, affect anthocyanins' yield and stability. Based on this experiment's results, it is clear that a proper extraction procedure for anthocyanins in foods with different matrixes must be devised. In frozen strawberry fruits, methanol and chloroform‐methanol produced the highest yield. The addition of MgO did not affect pigment degradation instead reduced the yield. The anthocyanin extraction in the pH differential method was lower than the methanol method, also produced haze, which developed small particles after storage and a freeze‐thaw process. The thawing process reduced the anthocyanin absorption, making the pH differential method unsuitable for strawberry anthocyanin assessment if the storage of the extract is needed. However, this method is the fastest method of anthocyanin assessment with reasonable anthocyanin recovery if time is a limiting factor or a large number of samples need to be tested. The method of Gauche et al. (2010) had longer processing time (a three‐step process). Both pH differential and Gauche et al. (2010) methods had lower anthocyanin yield than the methanol method and made them unsuitable for strawberry fruits. Further studies are needed to determine if other factors such as sample type (frozen, fresh, dried) or maceration methods affect the anthocyanin yield.

## CONFLICTS OF INTEREST

The authors declare that they do not have any conflict of interest.

## ETHICAL REVIEW

This study does not involve any human or animal testing.

## Data Availability

The data that support the findings of this study are available from the corresponding author upon reasonable request.

## References

[fsn32065-bib-0001] Akkarachaneeyakorn, S. , & Tinrat, S. (2015). Effects of types and amounts of stabilizers on physical and sensory characteristics of cloudy ready‐to‐drink mulberry fruit juice. Food Science & Nutrition, 3, 213–220. 10.1002/fsn3.206 25987996 PMC4431789

[fsn32065-bib-0002] Alexieva, V. , Sergiev, I. , Mapelli, S. , & Karanov, E. (2001). The effect of drought and ultraviolet radiation on growth and stress markers in pea and wheat. Plant, Cell and Environment, 24, 1337–1344. 10.1046/j.1365-3040.2001.00778.x

[fsn32065-bib-0003] Benchikh, Y. , Aissaoui, A. , Allouch, R. , & Mohellebi, N. (2020). Optimising anthocyanin extraction from strawberry fruits using response surface methodology and application in yogurt as natural colorants and antioxidants. Journal of Food Science and Technology, 18, 1–9. 10.1007/s13197-020-04710-0 PMC8021644 33897035

[fsn32065-bib-0004] Broeckling, C. , Li, K. G. , & Xie, D. Y. (2012). Comparative metabolomics of transgenic tobacco plants (*Nicotiana tabacum* var. Xanthi) reveals differential effects of engineered complete and incomplete flavonoid pathways on the metabolome: Çiftçi, Y. Transgenic Plants‐Advances and Limitations. InTech, 7, 379–396. 10.5772/32872

[fsn32065-bib-0005] Canuto, G. A. B. , Oliveira, D. R. , da Conceição, L. S. M. , Farah, J. P. S. , & Tavares, M. F. M. (2016). Development and validation of a liquid chromatography method for anthocyanins in strawberry (*Fragaria* spp.) and complementary studies on stability, kinetics and antioxidant power. Food Chemistry, 192, 566–574. 26304385 10.1016/j.foodchem.2015.06.095

[fsn32065-bib-0006] Desjardins, Y. (2008). Physiological and ecological functions and biosynthesis of health‐promoting compounds in fruit and vegetables. In F. A. Tomas‐Barberan , & M. I. Gil (Eds.), Improving the health‐promoting properties of fruit and vegetable products (pp. 201–247). Woodhead Publishing Series in Food Science, Technology and Nutrition.

[fsn32065-bib-0007] Favaro, L. I. L. , Balcão, V. M. , & Rocha, L. K. H. (2018). Physicochemical characterization of a crude anthocyanin extract from the fruits of Jussara (*Euterpe edulis* Martius): Potential for food and pharmaceutical. Journal of the Brazilian Chemical Society, 29, 2072–2088.

[fsn32065-bib-0008] Folch, J. , Lees, M. , & Sloane Stanley, G. H. (1957). A simple method for the isolation and purification of total lipids from animal tissues. Journal of Biological Chemistry, 226, 497–509. 13428781

[fsn32065-bib-0009] Gallik, S. (2012). Determination of the anthocyanin concentration in table wines and fruit juices using visible light spectrophotometry. Cell Biology, 2, 1–12.

[fsn32065-bib-0010] Garcia‐Viguera, C. , Zafrilla, P. , & Tomás‐Barberán, F. A. (1998). The use of acetone as an extraction solvent for anthocyanins from strawberry fruit. Journal of Plant, 9, 274–277. 10.1002/(SICI)1099-1565(199811/12)9:6<274:AID-PCA416>3.0.CO;2-GC

[fsn32065-bib-0011] Gauche, C. , Malagoli, E. S. , & Bordignon Luiz, M. T. (2010). Effect of pH on the copigmentation of anthocyanins from Cabernet Sauvignon grape extracts with organic acids. Scientia Agricola, 67, 41–46. 10.1590/S0103-90162010000100006

[fsn32065-bib-0012] Giusti, M. M. , & Wrolstad, R. E. (2001). Characterization and measurement of anthocyanins by UV‐Visible spectroscopy. Current Protocols in Food Analytical Chemistry. UNIT, F1.2–F1.2.13.

[fsn32065-bib-0013] Gu, X. , Cai, W. , Fan, Y. , Ma, Y. , Zhao, X. , & Chao, Z. (2018). Estimating foliar anthocyanin content of purple corn via hyperspectral model. Food Science & Nutrition, 6, 572–578. 10.1002/fsn3.588 29877500 PMC5980273

[fsn32065-bib-0014] Howard, L. R. , Brownmiller, C. , & Prior, R. L. (2014). Improved color and anthocyanin retention in strawberry puree by oxygen exclusion. Journal of Berry Research, 4, 107–116. 10.3233/JBR-140072

[fsn32065-bib-0015] Jayakumar, M. , Eyini, M. , Selvinthangadurai, P. , Lingakumar, K. , Premkumar, A. , & Kulandaivelu, G. (1999). Changes in pigment composition and photosynthetic activity of aquatic fern (*Azolla microphylla* Kaulf.) exposed to low doses of UV‐C (254 nm) radiation. Photosynthetica, 37, 33–38.

[fsn32065-bib-0016] Karaaslan, N. M. , & Yaman, M. (2017). Anthocyanin profile of strawberry fruit as affected by extraction conditions. International Journal of Food Properties, 20(sup3), S2313–S2322. 10.1080/10942912.2017.1368548

[fsn32065-bib-0017] Lee, J. , Durst, R. W. , & Wrolstad, R. E. (2005). Determination of total monomeric anthocyanin pigment content of fruit juices, beverages, natural colorants, and wines by the pH differential method: Collaborative study. Journal of AOAC International, 88, 1269–1278. 16385975

[fsn32065-bib-0018] Lindoo, S. J. , & Caldwell, M. M. (1978). Ultraviolet‐B radiation‐induced inhibition of leaf expansion and promotion of anthocyanin production: Lack of involvement of the low irradiance phytochrome system. Plant Physiology, 61, 278–282. 10.1104/pp.61.2.278 16660276 PMC1091848

[fsn32065-bib-0019] Liu, R. D. , Zhang, M. , & Li, X. X. (2008). Comparisons of extraction solvents and quantitative‐methods for analysis of anthocyanins in strawberry and blueberry fruits. Acta Horticulturae Sinica, 35(5), 655–660.

[fsn32065-bib-0021] Martín, J. , Kuskoski, E. M. , Navas, M. J. , & Asuero, A. G. (2017). Antioxidant capacity of anthocyanin pigments. In J. Justino (Ed.) Flavonoids: From biosynthesis to human health, (205–255). IntechOpen.

[fsn32065-bib-0022] Mazza, G. , Cacace, J. E. , & Kay, C. D. (2004). Methods of analysis for anthocyanins in plants and biological fluids. Journal of AOAC International, 87, 129–145. 15084096

[fsn32065-bib-0024] Neff, M. M. , & Chory, J. (1998). Genetic interactions between phytochrome A, phytochrome B, and cryptochrome during *Arabidopsis* development. Plant Physiology, 118, 27–36. 9733523 10.1104/pp.118.1.27 PMC34865

[fsn32065-bib-0025] Pavlidou, E. , Giaginis, C. , Fasoulas, A. , & Petridis, D. (2018). Clinical evaluation of the effect of blueberries consumption on chronic diseases, illness prevention and health promotion. The Natural Products Journal, 8(1), 45–53. 10.2174/2210315507666170830120953

[fsn32065-bib-0026] SAS Institute Inc (2015). SAS/IML® 14.1 User’s Guide. SAS Institute Inc.

[fsn32065-bib-0027] Silva, S. , Costa, E. M. , Calhau, C. , Morais, R. M. , & Pintado, M. E. (2017). Anthocyanin extraction from plant tissues: A review. Critical Reviews in Food Science and Nutrition, 57, 3072–3083. 10.1080/10408398.2015.1087963 26529399

[fsn32065-bib-0028] Solovchenko, A. E. , Chivkunova, O. B. , Merzlyak, M. N. , & Reshetnikova, I. V. (2001). A spectrophotometric analysis of pigments in apples. Russian Journal of Plant Physiology, 48, 693–700.

[fsn32065-bib-0029] Steele, M. R. , Gitelson, A. A. , Rundquist, D. C. , & Merzlyak, M. N. (2009). Non‐destructive estimation of anthocyanin content in grapevine leaves. American Journal of Enology and Viticulture, 60, 87–92.

[fsn32065-bib-0030] Taniguchi, M. , & Lindsey, J. S. (2018). Database of absorption and fluorescence spectra of >300 common compounds for use in photochem CAD. Photochemistry and Photobiology, 94, 290–327. 29166537 10.1111/php.12860

[fsn32065-bib-0031] Tonutare, T. , Moor, U. , & Szajdak, L. (2014). Strawberry anthocyanin determination by pH differential spectroscopic method‐ how to get true results. Acta Scientiarum Polonorum Hortorum Cultus, 13, 35–47.

[fsn32065-bib-0032] Trikas, E. D. , Papi, R. M. , Kyriakidis, D. A. , & Zachariadis, G. A. (2016). A sensitive LC‐MS method for anthocyanins and comparison of by‐products and equivalent wine content. Separations, 3(18), 1–12. 10.3390/separations3020018

[fsn32065-bib-0033] Zuiter, A. S. (2014). Proanthocyanidin: chemistry and biology: from phenolic compounds to proanthocyanidins. In J. Reedijk (Ed.) Reference module in chemistry, molecular sciences and chemical engineering, (1–29). Waltham, MA: Elsevier Inc.

